# *In Vitro* Modeling of the Neurovascular Environment by Coculturing Adult Human Brain Endothelial Cells with Human Neural Stem Cells

**DOI:** 10.1371/journal.pone.0106346

**Published:** 2014-09-04

**Authors:** Chung-Hsing Chou, John D. Sinden, Pierre-Olivier Couraud, Michel Modo

**Affiliations:** 1 University of Pittsburgh, McGowan Institute for Regenerative Medicine, Pittsburgh, Pennsylvania, United States of America; 2 Kings College London, Institute of Psychiatry, Department of Neuroscience, London, United Kingdom; 3 Tri-service General Hospital, Department of Neurology, National Defense Medical Centre, Taipei, Taiwan; 4 ReNeuron Ltd, Guildford, Surrey, United Kingdom; 5 INSERM U1016, Institut Cochin, Paris, France; 6 CNRS, UMR8104, Paris, France; 7 Université Paris Descartes, Sorbonne Paris Cité, Paris, France; 8 University of Pittsburgh, Department of Radiology, Pittsburgh, Pennsylvania, United States of America; 9 University of Pittsburgh, Department of Bioengineering, Pittsburgh, Pennsylvania, United States of America; Johns Hopkins University, United States of America

## Abstract

Brain and vascular cells form a functionally integrated signalling network that is known as the neurovascular unit (NVU). The signalling (autocrine, paracrine and juxtacrine) between different elements of this unit, especially in humans, is difficult to disentangle *in vivo*. Developing representative *in vitro* models is therefore essential to better understand the cellular interactions that govern the neurovascular environment. We here describe a novel approach to assay these cellular interactions by combining a human adult cerebral microvascular endothelial cell line (hCMEC/D3) with a fetal ganglionic eminence-derived neural stem cell (hNSC) line. These cell lines provide abundant homogeneous populations of cells to produce a consistently reproducible *in vitro* model of endothelial morphogenesis and the ensuing NVU. Vasculature-like structures (VLS) interspersed with patches of differentiating neural cells only occurred when hNSCs were seeded onto a differentiated endothelium. These VLS emerged within 3 days of coculture and by day 6 were stabilizing. After 7 days of coculture, neuronal differentiation of hNSCs was increased 3-fold, but had no significant effect on astrocyte or oligodendrocyte differentiation. ZO1, a marker of adherens and tight junctions, was highly expressed in both undifferentiated and differentiated endothelial cells, but the adherens junction markers CD31 and VE-cadherin were significantly reduced in coculture by approximately 20%. A basement membrane, consisting of laminin, vitronectin, and collagen I and IV, separated the VLS from neural patches. This simple assay can assist in elucidating the cellular and molecular signaling involved in the formation of VLS, as well as the enhancement of neuronal differentiation through endothelial signaling.

## Introduction

The neurovascular unit (NVU) is the quintessential organizational principle of functional brain tissue [1]. Signaling between the vascular and neural cells is a key physiological process regulating the interaction between peripheral circulation and brain activity. At the interface of the vascular and neural compartments is the blood-brain barrier (BBB) that limits the access of molecules and peripheral cells to the brain. In the healthy brain, neuronal and glial activity will influence vascular function by neurovascular coupling to regulate their energetic demands by maintaining tissue oxygenation and nutrient influx [2]. In the case that this energetic demand is not met, a rapid physiological response to hypoxia (i.e. lack of sufficient oxygenation) induces an angiogenic cascade that aims to restore tissue oxygenation [3]. Angiogenesis, a growth of new vessels from existing vessels, increases the vascular density and warrants adequate tissue oxygenation. In brain tumors, the continued growth of the “neural” compartment is perpetuating a state of mild hypoxia, hence producing an ongoing angiogenesis [4]. In contrast, in stroke, where blood flow to brain tissue is blocked for protracted periods of time, cells will die. Nevertheless, peri-infarct regions undergo a mild hypoxia engendering a vascular response encompassing vasculogenesis, angiogenesis, arteriogenesis and collateral growth [5]. This leads to heteregeneous peri-infarct areas of neo- and hypervascularization [6].

In the peri-infarct regions of a stroke, a local increase in neovascularization is also observed after implantation of neural stem cells (NSCs) [7], [8] and has been suggested by some to mediate behavioral improvements [9], [10]. It is conceivable that these cells have a propensity to directly act on the host vasculature, but the increase of energetic demand in the area of implantation due to an increase of cellular density can also induce mild hypoxia that promotes neovascularization. It is important to note that NSCs injected into a stroke cavity with bioscaffolding by themselves do not lead to re-vascularization [11], [12], a common problem observed in tissue engineering [13], [14]. Strategies aimed at modulating the vascular response following a stroke [15] or NSC transplantation [9], [10], vascularization of engineered tissues [16], as well as inhibition of angiogenesis in brain tumors [17] are grounded on a thorough understanding of the dynamic paracrine, autocrine and juxtacrine interactions in the NVU [18], [19]. However, parceling out these interactions *in vivo*, especially in humans, is extremely challenging. Therefore the development of *in vitro* models that afford a high level of experimental control and reproducibility is required to complement *in vivo* studies [20].

Although a variety of *in vitro* models have been developed to investigate angiogenesis [21] and the BBB [22], as well as the NVU using rodent cells [23], assays composed of only human cells investigating the formation of vasculature-like structures (VLS) of brain microvascular endothelial cells by interacting with brain cells, i.e. establishing a neurovascular environment, are generally lacking. Although co-culturing of hNSCs with non-CNS endothelial cells, such as Human Umbilical Vein Endothelial Cells (HUVECs), have been reported to identify paracrine factors that promote hNSCs proliferation [24], as well as angiogenesis-promoting factors [25], these do not model the interaction between hNSC and brain endothelium. Transwell experiments only investigate paracrine and autocrine factors, whereas extracellular matrix models, such as the Matrigel assay, are predominantly biased towards juxtacrine factors [26], hence these are more suitable to investigate a specific type of factors rather than their synergistic and iterative effects. However, given the appropriate conditions, endothelial cells will organize *in vitro* into a net of VLS, akin to a vascular bed in tissue. An *in vitro* model of the neurovascular environment therefore needs to involve direct cell-to-cell contact between endothelial and “brain” cells, while forming a network of VLS, hence affording the investigation of the dynamic interactions of the formation of a neurovascular environment, as well as the processes involved in angiogenesis in the brain.

We here describe an all-human *in vitro* model that affords controlled and detailed investigations of interactions between previously validated brain microvascular endothelial and neural stem cell lines, while forming a network of VLS. The resulting cytoarchitecture is reminiscent of the neurovascular environment with endothelial cells organized into a vasculature-like structure surrounded by astrocytes, neurons and oligodendrocytes.

## Materials and Methods

### Human neural stem cell (hNSC) line

The derivation of the c-mycER^TAM^ transduced hNSC line STROC05 (ECACC accession number 04110301, provided by ReNeuron Ltd., Surrey, UK) has been previously described [27]. In brief, hNSCs were isolated from the whole ganglionic eminence of a human fetus at 12 weeks of gestation. The cells were transduced with the retroviral vector pLNCX-2 (Clontech) encoding the c-mycER^TAM^ gene [28]. Expansion and maintenance of STROC05 cells were performed in tissue culture flasks (BD Biosciences) coated with mouse laminin (Sigma-Aldrich, L2020) at a concentration of 10 µg/ml at 37°C in 5% CO_2_. STROC05 cells were cultured in serum-free medium consisting of DMEM:F12 medium (Sigma) containing 5 µg/ml insulin (Sigma), 100 µg/ml transferrin (Sigma), 40 ng/ml sodium selenite (Sigma), 60 ng/ml progesterone (Sigma), 16.2 µg/ml putrescine (Sigma), 0.03% human albumin solution (GemBio), 400 ng/ml L-thyroxine (Sigma), 337 ng/ml tri-iodo-thyronine (Sigma), 10 units/ml heparin sodium (Sigma), 40 ng/ml corticosterone (Sigma), and 2 mM L-glutamine (Sigma) (Table S1). Recombinant human basic fibroblast growth factor (bFGF; 10 ng/ml; PeproTech), epidermal growth factor (EGF; 20 ng/ml; PeproTech), and 4-hydroxytamoxifen (100 nM; Sigma) were added as mitogens. The cells were passaged and used for experiments when they reached 70–80% confluency.

### Human cerebral microvascular endothelial cell (hCMEC/D3) line

The immortalized human cerebral microvascular endothelial cell line (hCMEC/D3) was isolated from microvessel fragments of the temporal lobe of an adult with epilepsy by coexpressing human telomerase reverse transcriptase and simian vacuolating virus 40 (hTERT/SV40) large T antigen via a lentiviral vector transduction system [29]. This cell line has been widely used as an *in vitro* model of human brain endothelium [30]. The cell line was expanded and maintained in tissue culture flasks (BD Biosciences) coated with rat tail collagen type I (BD Biosciences, 354236) at a concentration of 150 µg/ml at 37°C in 5% CO_2_, using endothelial basal medium-2 (EBM-2; Lonza), supplemented with 5% fetal bovine serum “Gold” (PAA, The Cell Culture Company), 5 µg/ml ascorbic acid (Sigma), 1% chemically defined lipid concentrate (Invitrogen), 10 mM HEPES buffer (Sigma), and 1% penicillin/streptomycin (Invitrogen) (Table S1). hCMECs were used between passages 28 and 32. Cells were passaged at 95% confluency.

### Endothelial morphogenesis – Matrigel assay

The Matrigel (BD Biosciences, 356237) endothelial branching morphogenesis assay establishes the potential of ECs to form tubular networks (also known as capillary-like structures, CLS) [31]. For this, 300 µl Matrigel (10.4 mg/ml, not diluted) was added to the wells of 24-well plates and allowed to gel at 37°C for 30 minutes. Then 40,000 hCMECs were added to each well and allowed to invade the material for 48 hours. As a positive control for this assay, an immortalized microvascular EC line (HMEC-1) derived from human foreskins was used [32]. HMEC-1 cells were expanded in MCDB131 (Sigma), supplemented with 10% fetal calf serum (Sigma), and 1% penicillin/streptomycin (Invitrogen).

**Figure 1:**
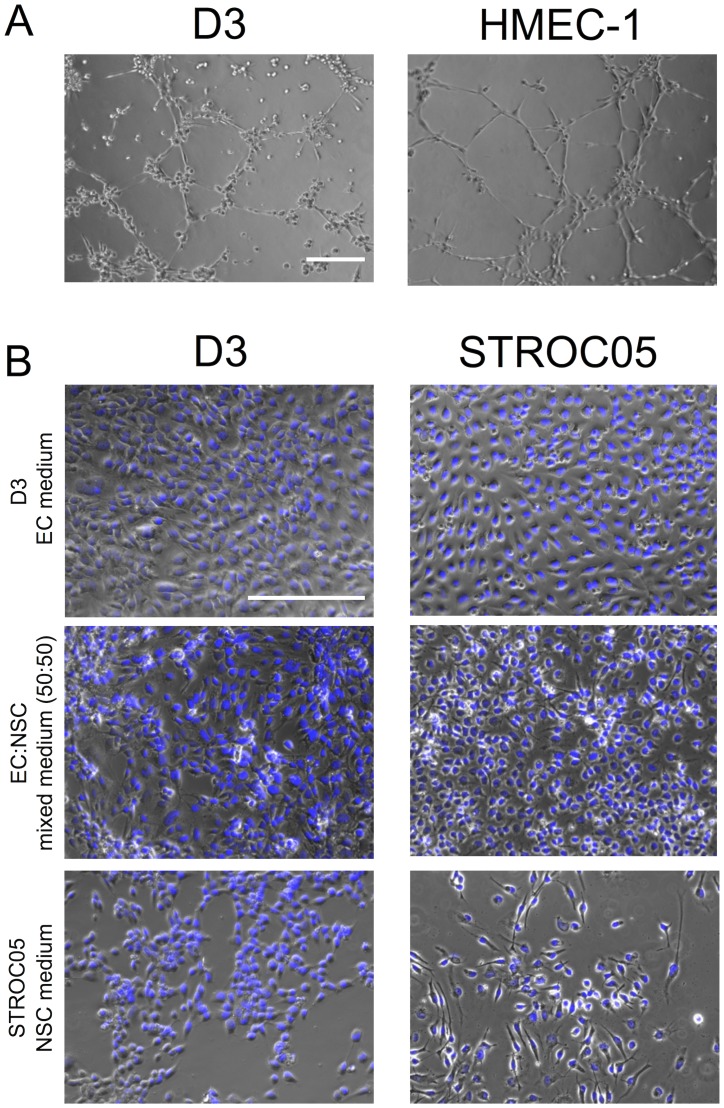
Establishing branching phenotype of endothelial cells and media for coculture with neural stem cells. (A) Matrigel branching morphogenesis assay confirmed the potential for endothelial morphogenesis in D3 human cerebral microvasculature endothelial cells (hCMECs) and HMEC-1 dermal EC lines. (B) D3 hCMECs in EC medium formed a cobblestone-like monolayer on collagen-coated surfaces. D3 hCMECs in NSC medium started to detach from culture surfaces, and STROC05 human neural stem cells (hNSCs) in EC medium lost bipolar elongated morphology. In comparison, morphology of D3 hCMECs and STROC05 hNSCs was maintained in a 50∶50 mix of EC:NSC medium. Diamidino-2-phenylindole (DAPI, blue) serves as a nuclear counterstain. Scale bars represent 200 µm.

The Matrigel endothelial morphogenesis assay confirmed the branching phenotype in both hCMEC and HMEC-1 lines (Fig. 1A). hCMECs therefore have the potential for endothelial morphogenesis, but when cultured on a collagen-coated surface, hCMECs are organized in an extensive and homogeneous cobblestone-like monolayer displaying a brain microvascular morphology with tightly packed elongated cells (Fig. 1B), as well as characteristics of the BBB [29].

### *In vitro* modeling of the neurovascular unit – Culture media

D3 hCMECs and STROC05 hNSCs utilize medium of different composition. To coculture these, it is therefore essential to determine how these different media or their combination affect cell morphology and culture consistency (e.g. attachment characteristics, density). Culture media specific to D3 (EC medium) or STROC05 cells (NSC medium) were used in monoculture to expand cells until they reached confluency on coverslips (VWR, 89015-724) coated either with collagen I (hCMECs) or laminin (hNSCs) in 24-well plates. Media were then replaced every other day with either EC or NSC medium without addition of mitogens and tamoxifen (i.e. differentiation media), or a 50∶50 mix of EC:NSC media (Table S1). Cell morphology was evaluated 7 days later. EC medium resulted in both D3 hCMECs and STROC05 hNSCs adapting a dense cobblestone morphology without extension of filopodia (Fig. 1B). In contrast, NSC medium lead to detachment of hCMECs and reduced density and cell differentiation. The 50∶50 media mix provided the most consistent cell morphology of both cell lines, while preserving robust attachment and cellular density. hNSCs preserved their ability to extend filopodia in the mixed medium. The EC:NSC serum-free coculture media mix therefore retained both hCMEC and hNSC features and was consequently used for all coculture experiments.

### *In vitro* modeling of the neurovascular unit – Experimental set-up

**Figure 2:**
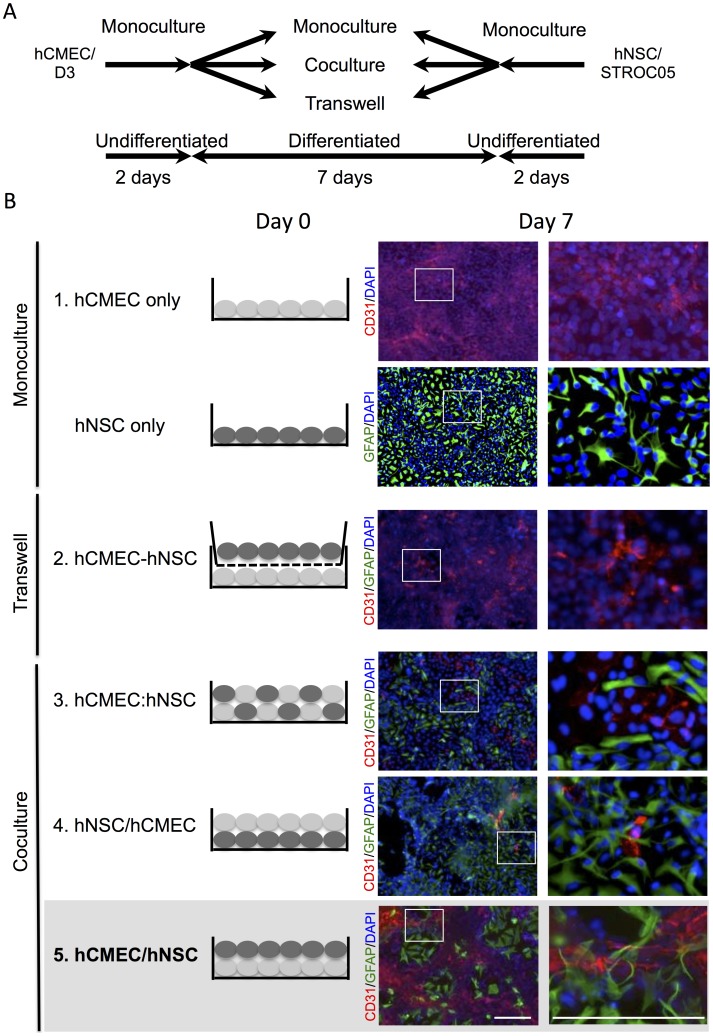
Protocols for EC/NSC coculture. (A) Schematic description of the protocols for monoculture and coculture of D3 human cerebral microvascular endothelial cells (hCMECs) and STROC05 human neural stem cells (hNSCs). (B) 1. hCMECs (CD31+ cells) formed a dense monolayer on the collagen-coated surface, but did not form vasculature-like structures (VLS). hNSCS (polyclonal GFAP+ cells) did not form any VLS. 2. In a transwell coculture, only a dense monolayer of hCMECs without significant VLS could be found at the bottom of the lower chamber after 7 days of coculture. 3. No significant VLS was found when hCMECs were seeded in combination with hNSCs. 4. No VLS emerged when hCMECs were seeded on 7-day differentiated STROC05 cells. 5. A distinctive cytoarchitecture composed of CD31+ VLS and GFAP+ cells was observed when hNSCs were seeded on 7-day differentiated hCMECs for a further 7 days of coculture. Diamidino-2-phenylindole (DAPI, blue) serves as a nuclear counterstain. Scale bars represent 200 µm.

A major advantage of *in vitro* models over *in vivo* models is the greater experimental control over the cellular and molecular interactions being investigated. The use of two cell lines here provides an opportunity to study various aspects of EC-NSC interaction ranging from how particular molecules affect a single cell type (monoculture), to the effects of secreted (paracrine & autocrine) factors in a transwell assay, as well as the interaction between differentiated and undifferentiated cells when these are cocultured (paracrine, autocrine & juxtacrine factors combined). Typically, 7 days of coculture are sufficient to investigate these dynamic interactions (Figure 2A). The following conditions have been investigated here (Fig. 2B):

#### Monoculture – modeling cell line specific features

To investigate hCMECs or hNSCs interactions, hCMECs (40,000 cells) or hNSCs (25,000 cells) were grown in monoculture in a 24-well plate using 50∶50 EC:NSC coculture media (500 µl). Glass coverslips (VWR, 89015-724) in the wells were coated either with collagen I or laminin. This condition provides a key comparison to establish how cellular signaling and behavior within single cell type is influenced by the presence of specific molecules (secreted and/or juxtacrine). It therefore provides an important control condition compared to conditions where different cell types are cultured together. Culture medium was replaced every other days and cells were fixed after 7 days with 4% paraformaldehyde (PFA) or 95% ethanol for 10 minutes, rinsed with phosphate buffered saline (PBS), and stored in PBS at 4°C.

#### hCMEC–hNSC transwell assay – modeling the influence of secreted factors only

The use of a transwell allows secreted (autocrine & paracrine) factors to distribute in the culture media and influence cell behavior without the contribution of juxtacrine factors. Here, transwell inserts (Transwell with 0.4 µm pore polyester membrane insert, Corning, 3470) were coated with mouse laminin (Sigma-Aldrich, L2020) at a concentration of 10 µg/ml at 37°C in 5% CO_2_ for 2 h. Then STROC05 cells were seeded into transwell inserts at a density of 5,000 cells per 0.33 cm^2^ area, for proliferation to 80% confluency. Meanwhile, D3 cells, with a seeding density of 40,000 cells/well, were grown separately on collagen-coated coverslips in 24-well plates for 7 days. The transwell inserts containing STROC05 cells were then transferred to the wells containing D3 cells, and these were cultured together in serum-free coculture medium. The distance between the transwell membrane and the bottom of the monolayer of D3 cells was about 850 µm.

#### hCMEC:hNSC coculture of undifferentiated cells – modeling the effects of proliferating cells on each other

To model the interactions between hCMECs and hNSCs, while both are proliferating and undifferentiated, 40,000 D3 cells and 25,000 STROC05 cells were combined and seeded together onto glass coverslips (VWR, 89015-724) coated with a mixture of 150 µg/ml collagen I and 10 µg/ml laminin in 24-well plates, using serum-free coculture medium which did not contain mitogens or tamoxifen to promote the continued proliferation of hNSCs.

#### hNSC/hCMEC – modeling the effects of proliferating endothelial cells on brain tissue

To model how proliferating hCMECs affect brain cells, undifferentiated hCMECs (40,000 cells/well) were added to hNSC cultures (seeded at a density of 25,000 cells/well) that had been differentiated on laminin-coated coverslips in 24-well plates for 7 days when there was an absence of proliferating cells.

#### hCMEC/hNSC – modeling the effects of hNSCs implants on host vasculature

To model the interactions hNSCs exert on the host brain vasculature, undifferentiated hNSCs were added to 7-day differentiated D3 cells. For this, D3 (40,000 cells/well) were seeded on glass coverslips (VWR, 89015-724) coated with rat tail collagen type I (BD Biosciences), using D3 EC medium, in 24-well plates for 7 days. STROC05 (25,000 cells/well) were added on day 8 in the presence of coculture medium. Only this condition formed “spontaneous” VLS (Figure 2B), hence indicating that the dynamic interactions between differentiated hCMECs and undifferentiated hNSCs provided the sufficient signaling conditions for novel neurovascular units to form. This condition was further characterized as an assay to investigate how hNSCs induce the formation of VLS by hCMECs.

### Time course of vasculature-like structure formation

To establish a time course of VLS formation, hCMECs were seeded (40,000 cells/well) and allowed to differentiate for 7 days before hNSCs (25,000 cells/well) were added (hCEMC/hNSC condition) and cocultured for 7 days. To investigate cytoarchitectural changes resulting from cell movements, a series of time-lapse images were photographed under a digital inverted microscope (EVOS f1, AMG) by phase imaging. Coverslips were fixed each day with 4% PFA, as described above.

### Immunocytochemistry and image acquisition

For immunocytochemistry, cells were blocked with 10% normal goat serum in PBS containing 0.1% Triton X-100 (Sigma) for 30 min prior to incubation with primary antibodies for 18 h at 4°C. The mouse anti-glial fibrillary acid protein (GFAP) (1∶3000; Sigma, G3893) specifically detected the astrocytic differentiation of hNSC in vitro (<10% of undifferentiated cells were positive), whereas the polyclonal chicken anti-glial fibrillary acid protein (GFAP) (1∶3000; Abcam, ab4674) was unspecific, detecting almost 100% of undifferentiated and >80% of differentiated hNSCs. The polyclonal antibody was therefore used here to distinguish vascular from neural compartments, whereas the monoclonal GFAP was used to determine differentiation. Either the rabbit (1∶200; Abcam, ab28364) or mouse (1∶200; Abcam, ab119339) anti-CD31/platelet endothelial cell adhesion molecule 1 primary antibodies were used to visualize endothelial cells representing the “vascular component”. Additionally, mouse anti-microtubule associate protein-2 (MAP2) (1∶500; Abcam ab11267), mouse anti-galactocerebroside (GalC) (1∶200; Millipore MAB342), mouse anti-vascular endothelial-cadherin (VE-cad) (1∶200; Abcam ab7047), rabbit anti-zonula occludens 1 (ZO1) (1∶500; Zymed 40-2200), rabbit anti-occludin (1∶200; Abcam ab64482), rabbit anti-claudin-5 (1∶200; Abcam ab53765), mouse anti-intercellular adhesion molecule 2 (ICAM-2) (1∶200; Santa Cruz sc-9987), mouse anti-podocalyxin-like 1 (1∶200; Santa Cruz sc-23904), chicken anti-laminin (1∶500; Abcam ab14055), and goat anti-collagen IV (1∶200; Millipore ab769) primary antibodies were used for investigating differentiation status of monoculture and coculture. After removal of primary antibodies and washing with PBS (3x), cells were incubated with appropriate secondary antibodies (goat anti-mouse Alexa 488-labeled, 1∶1000; goat anti-rabbit Alexa 555-labeled, 1∶1000, Invitrogen) for 1 h at room temperature (22°C). Stained coverslips were rinsed in PBS and mounted with Vectashield for fluorescence with DAPI (Vector Laboratories). Fluorescence images were captured using a fluorescence microscope (AxioImager M2, Zeiss), a confocal laser-scanning microscope (Fluoview 1000, Olympus), and a digital inverted microscope (EVOS f1, AMG).

**Figure 3:**
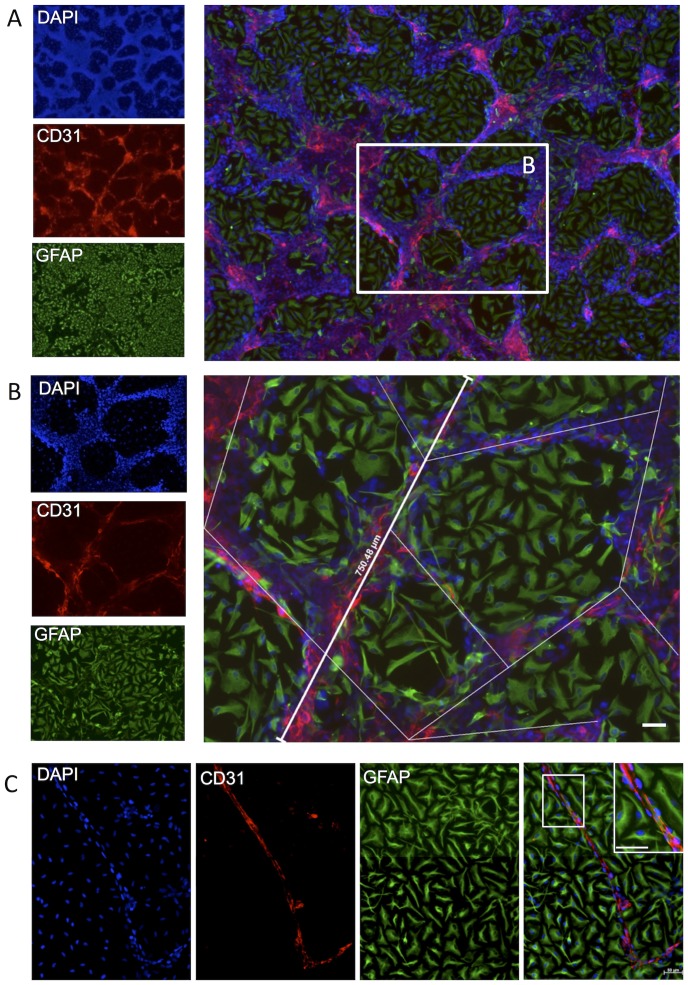
Immunohistochemistry of junctional markers on endothelial cells. Localization of ZO1 expression/detection was heterogeneous, with it being only visible as part of the membrane (i.e. cellular junctions) on elongated and arranged endothelial cells (red arrows), but being mostly cytoplasmic in clumped and non-elongated cells (yellow arrows). As the cytoplasmic expression is decreased (grey arrow), it gradually shifts towards being exclusively present in the membrane.

Expression of endothelial markers, especially junctional proteins, such as CD31, VE-cadherin, ZO1, ICAM-2, occludin, claudin-5, exhibit a heterogeneous appearance that can reflect typical in vivo localization of these markers at cellular junctions, but can also reflect a cytoplasmic expression (Figure 3). The cytoplasmic localization of these molecules is untypical of the in vivo expression pattern of these markers on endothelial cells. It is hence important to note that merely demonstrating the presence of these proteins is not indicative of functional tight junctions.

### Measuring the length of vasculature-like structure

**Figure 4:**
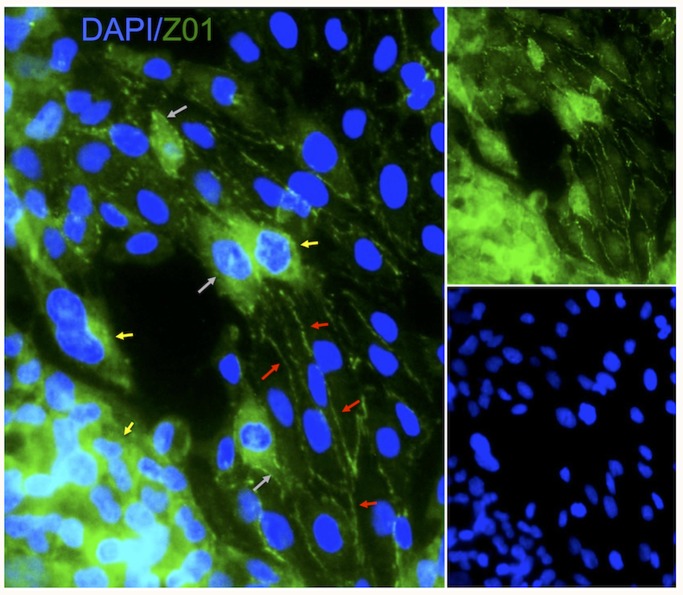
Quantification of endothelial morphogenesis. (A) A distinctive neurovascular cytoarchitecture emerged in which hCMECs (CD31+) formed vasculature-like structures (VLS) resembling a vascular network in between patches of hNSCs (polyclonal GFAP+). (B) The efficiency of VLS formation was quantified by measuring the length of segments between VLS branching points. (C) In a few samples, singular capillary-like structures comprising single layers of CD31+ cells between which a lumen-like space formation was observed. Diamidino-2-phenylindole (DAPI, blue) serves as a nuclear counterstain. Scale bars represent 50 µm.

To determine the efficiency of forming VLS (Figure 4A), quantification of the formation of VLS was achieved using the Stereo Investigator (MBF Bioscience) software. The extent of VLS formation was evaluated by measuring the length of segments between branching points (Figure 4B), as well as counting the number of branching points. The total length of segments, as well as the number of branching points were calculated to compare the efficiency of VLS formation [33]. A few capillary-like segments comprised a single layer of CD31+ cells between which a lumen-like space formation were observed (Fig. 4C), but were not representative of the overall formation of VLS.

### Statistics

All experiments consisted of 3 biological replicates, each consisting of 3 technical replicates. For each technical replicate, 5 images were taken from different areas on the coverslip prior to calculating a mean value of cell counts for each coverslip. Using SPSS 17 for Mac (IBM), a non-parametric Kruskall-Wallis was used to compare different conditions followed by a Dunn’s post-hoc comparison to determine which conditions were significantly different (p<0.05). A Mann-Whitney U test was used to compare the markers of differentiation between mono- and cocultures. Graphs were drawn in Prism 5 (GraphPad) with data points representing the median and bars reflecting the value range.

## Results

### Vasculature-like structures only form in direct contact between differentiated endothelium and undifferentiated neural stem cells

Monocultures of hCMECs on collagen I form a homogenous cobblestone-like monolayer of cells, but do not organize into VLS (Figure 2B). The organization of hCMECs into *in vitro* tubular structures is therefore lacking the appropriate signaling. In the NVU, endothelial cells interact with brain cells to form new vessels. Therefore co-culturing these will allow us to determine what signaling interactions are key to the organization of hCMECs into new VLS. Secreted factors can be selectively investigated by using a transwell approach, but these did not provide sufficient signaling for hCMECs to form VLS (hCMEC-hNSC condition in Figure 2B). As hCMECs form CLS in Matrigel, contact-mediated factors, in addition to those secreted from hNSCs, might therefore be required to form VLS. Combining undifferentiated hCMECs and hNSCs or differentiated hNSCs with undifferentiated hCMECs resulted in the formation of some VLS, although these did not organize into a net-like connected structure. hCMECs only efficiently formed VLS and net-like structures when first differentiated and combined with undifferentiated hNSCs, indicating that specific signaling conditions are required to induce a vasculogenic process and that secreted factors by themselves are insufficient to induce this process.

**Figure 5:**
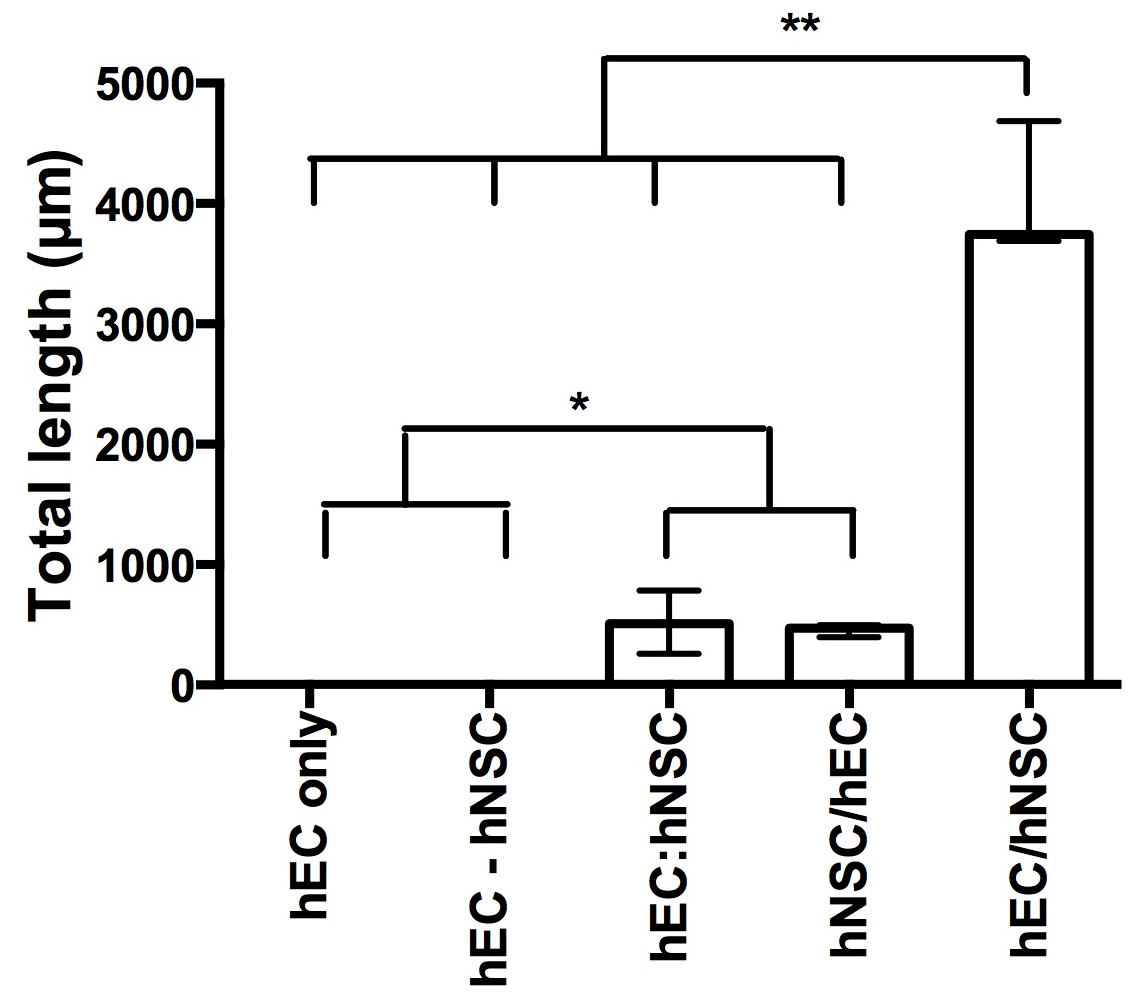
Comparison of efficiency to form vasculature-like structures. Total length of VLS in hCMEC/hNSC coculture was more efficient than any of the other culture conditions, including hCMEC monoculture (hCMEC only), the transwell coculture (hCMEC-hNSC), seeding of hCMECs and hNSCs simultaneously (hCMEC:hNSC), and seeding hCMECs on differentiated hNSCs (hNSC/hCMEC). Data points on the graph represent the median with bars reflecting the value range (post-hoc pairwise comparisons: * p<.05; **p<.01).

The co-culture of initially juxtaposed monolayers of differentiated hCMECs and undifferentiated hNSCs resulted in a major reorganization of cells. hCMEC organized into a vasculature-like structure that formed a network interspersed by neural patches, akin to the appearance of a neurovascular tissue (Figure 4A). To assess the efficiency of the formation of VLS, the length of individual segments of the network of VLS were measured (Figure 4B). There was also occasionally evidence of the formation of capillary-like structures (Figure 4C) that in contrast to VLS were much thinner (1–2 endothelial cells in width), but these were unrepresentative and only found in areas where endothelial cells were mostly absent. The quantification of VLS segment length supported the qualitative observation of VLS formation with no VLS forming in the EC only or transwell condition, but some VLS forming in contact-medicated co-culture conditions (undifferentiated hCMEC and hNSC intermixed, differentiated hNSCs with undifferentiated hCMEC seeded on top) (Figure 5, Kruskall Wallis = 13.04, p<.01). However, these VLS formed very inefficiently compared to undifferentiated hNSC being seeded on top of differentiated hCEMC. An almost 8 fold increase in efficiency could be observed to the other contact co-culture conditions.

### Cytoarchitectural characterization of vasculature-like structures

**Figure 6:**
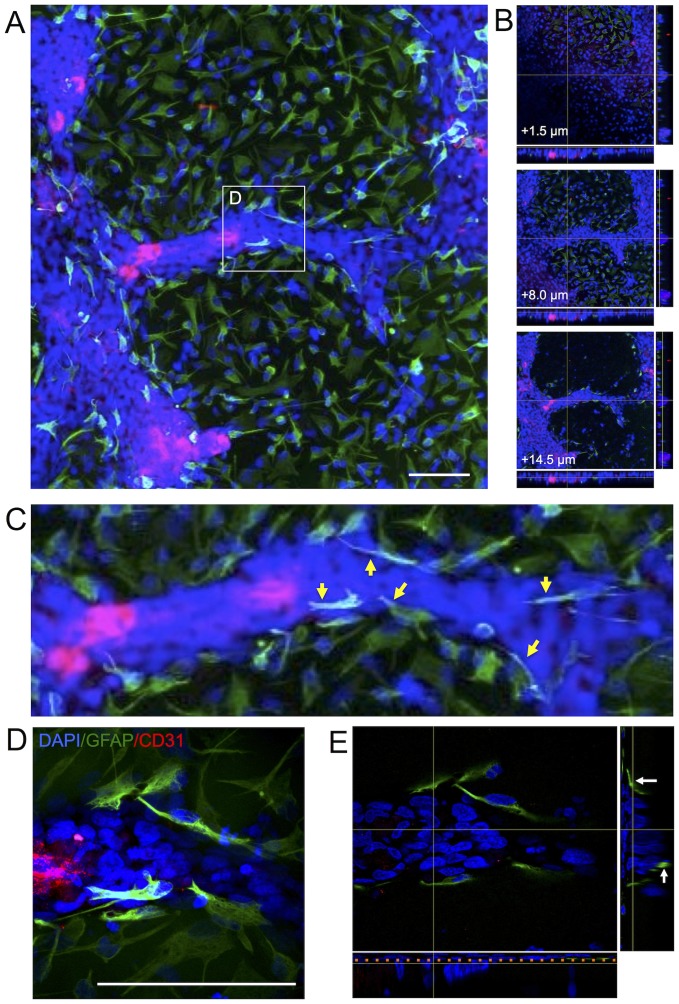
3-dimensional cytoarchitecture of vasculature-like structures. (A) A 3-dimensional cytoarchitecture composed of hCMECs (CD31+ cells in red) and hNSCs (polyclonal GFAP+ cells in green) was visualized using confocal microscopy. (B) At the base of the coculture, hNSCs were seen in a monolayer on which hCMECs formed VLS that was characterized by a multicellular organization that extended beyond the monolayer to form a unique structure. (C) Some hNSCs with an astrocytic phenotype were associated with these VLS by forming a layer of cells around the tubular walls in some cases with an elongated morphology and endfeet on the VLS (yellow arrows). The astrocytes were ensheathing the VLS. (D) A higher magnification 3D confocal image clearly demonstrates structural differences in astrocytes’ morphology upon interfacing with the VLS rather than being inside the neural patch. (E) A 3-dimensional cut through this area further indicates that astrocytes provide structural support (white arrows) to the VLS. The orange line indicates the monolayer formed by hNSCs onto which the VLS rests. Nevertheless, no hollow lumen is formed by the VLS. It hence here remains unclear if a 3-dimensional support structure is required to create a lumen and if they are inflatable in a flow system. Diamidino-2-phenylindole (DAPI, blue) serves as a nuclear counterstain. Scale bars represent 100 µm.

Upon co-culturing of hNSCs with differentiated hCMEC, hCMEC organize into a 3 dimensional structure of 8–10 hCMECs in width (Figure 6A) with diameters ranging from 8–50 µm. hNSCs cover the coverslip with hCMECs assembling and rising above these into VLS (Figure 6B). Average length of VLS was approximately 300 µm on day 7, but some longer structures up to 800 µm were also observed. hNSCs differentiated into astrocytes (GFAP+ cells) overlying the 3 dimensional VLS (Figure 6C&D), a specific anatomical feature of microvessels in the brain. Nevertheless, the VLS did not form a hollow lumen (Figure 6E).

**Figure 7:**
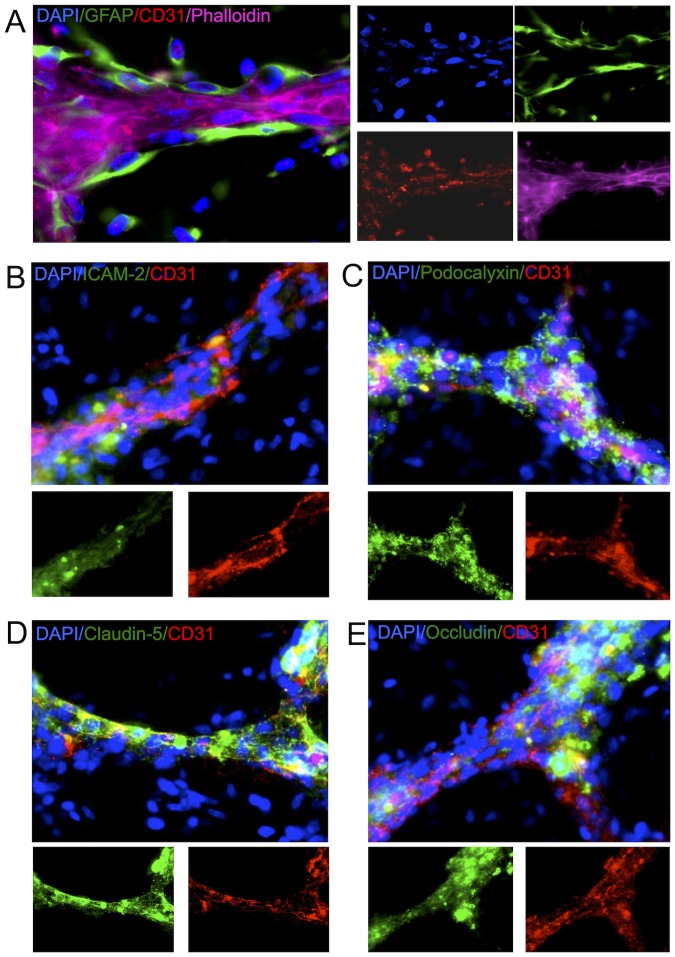
Cytoarchitectural characterization of vessel-like structures. (A) Astrocytes (monoclonal GFAP+ cells) form a layer of support around endothelial cells that organize into tubular structures readily identified by phalloidin. (B) Tubular structures are tightly packed with endothelial cells expressing intercellular adhesion molecule-2 (ICAM-2), although there is a degree of inconsistency in along the VLS indicating different stages of development/maturity. (C) Endothelial cells organized into VLS are polarizing as indicated by the expression of the apical marker podocalyxin that influences astrocytes positioning of endfeet. (D) Endothelial cells within the VLS also express markers indicative of tight junctions, such as Claudin-5 and Occludin.

VLS were clearly distinguishable based on their cellular density and presence of CD31, an intercellular junction marker present in endothelial cells, with astrocytes tightly bound around these (Figure 7A). Nevertheless, CD31 was mostly localized within the cytoplasm rather than at the intercellular junction typically observed in endothelial cells in vivo. Only VLS expressed the intercellular adhesion molecule 2 (ICAM-2) indicating ongoing vessel formation, although parts of the VLS no longer expressed ICAM-2 (Figure 7B). Conversely, parts of the VLS that appeared more robust expressed Podocalyxin on the apical aspect of the structure (Figure 7C), although these are devoid of a lumen. Expression of Claudin-5 (Figure 7D) and Occludin (Figure 7E) is evident, but is localized mostly inside the cells rather than the pints of cell-cell contact. Nevertheless, the presence of these junctional proteins by themselves is not an indication that functional tight junctions are being formed in these VLS.

**Figure 8:**
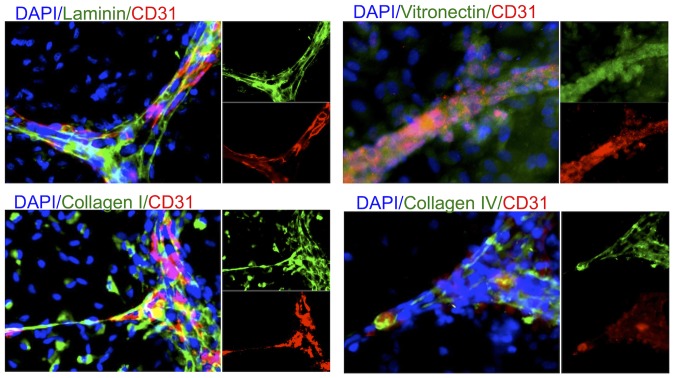
Deposition of a basement membrane and presence of tight junction molecules. Characteristic extracellular matrix molecules, such as laminin, vitronectin, collagen I and IV, delineate the basement membrane which separates the vascular-like structures from the neural environment.

A further cytoarchitectural feature of the neurovascular unit is the formation of a basement membrane that separates the vascular structure from the neural tissue. The non-apical aspect of the VLS presented the characteristic basement membrane molecules laminin, vitronectin, as well as collagen I & IV (Figure 8), further indicating that the VLS are forming vascular elements of the NVU. Collagen I was also expressed in approximately 50% of non-endothelial cells, whereas the other 3 extracellular membrane molecules were only found within the VLS.

### Neurovascular morphogenesis is a gradual process

**Figure 9:**
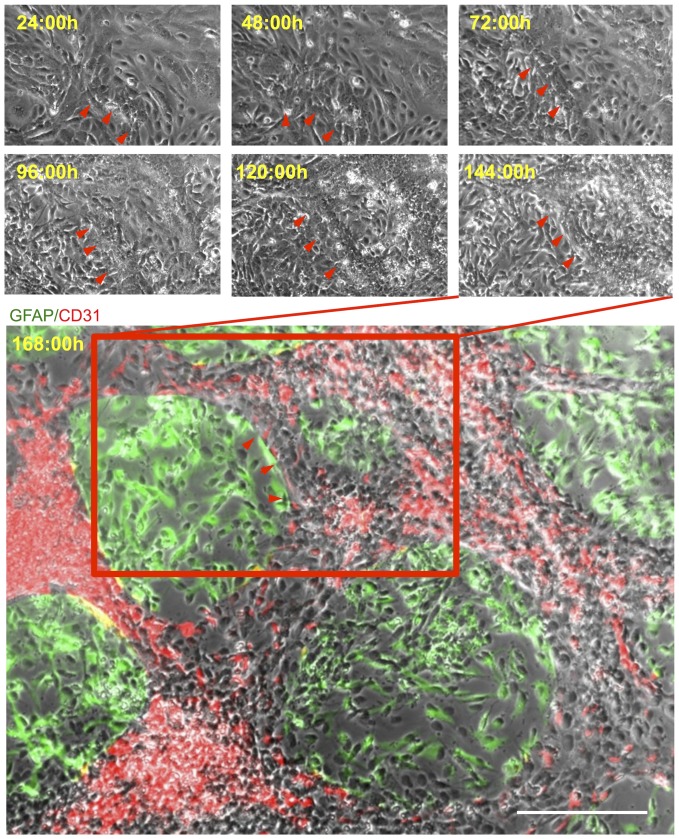
Time-lapse of vasculature-like structure formation. Time-lapse images revealed the gradual development of a neurovascular cytoarchitecture in hCMEC/hNSC coculture. Arrowheads indicate the border of VLS that became prominent after coculture for 72 h. This solid cytoarchitecture was fixed after an observation period of 168 h. The contrast at borders between hCMECs (CD31+ in red) and hNSCs (polyclonal GFAP+ in green) areas was enhanced computationally. Diamidino-2-phenylindole (DAPI, blue) serves as a nuclear counterstain. Scale bar, 200 µm.

To visualize the process of endothelial morphogenesis leading to the formation of VLS, a series of time-lapse images of hCMEC/hNSC coculture were taken every 24 hrs for 7 days (Figure 9). These images revealed that hCMECs responded to the presence of hNSCs by increasing their spatial density through alignment, as well as by adopting an elongated shape. This endothelial morphogenesis, defining the emergence of VLS, was evident within 72 hrs of coculture. In contrast, hNSCs infiltrated the vacant space and further formed patches that increasingly defined the VLS by providing mural support for hCMEC stacking. On day 7 (168 hrs), the endothelial and neural space is distinctly defined into two separate compartments. This time line indicates that hCMECs adapt from a differentiated phenotype adhering to the tissue culture substrate to preferentially adhering to other endothelial cells and providing a substrate for adhesion of some hNSCs.

**Figure 10:**
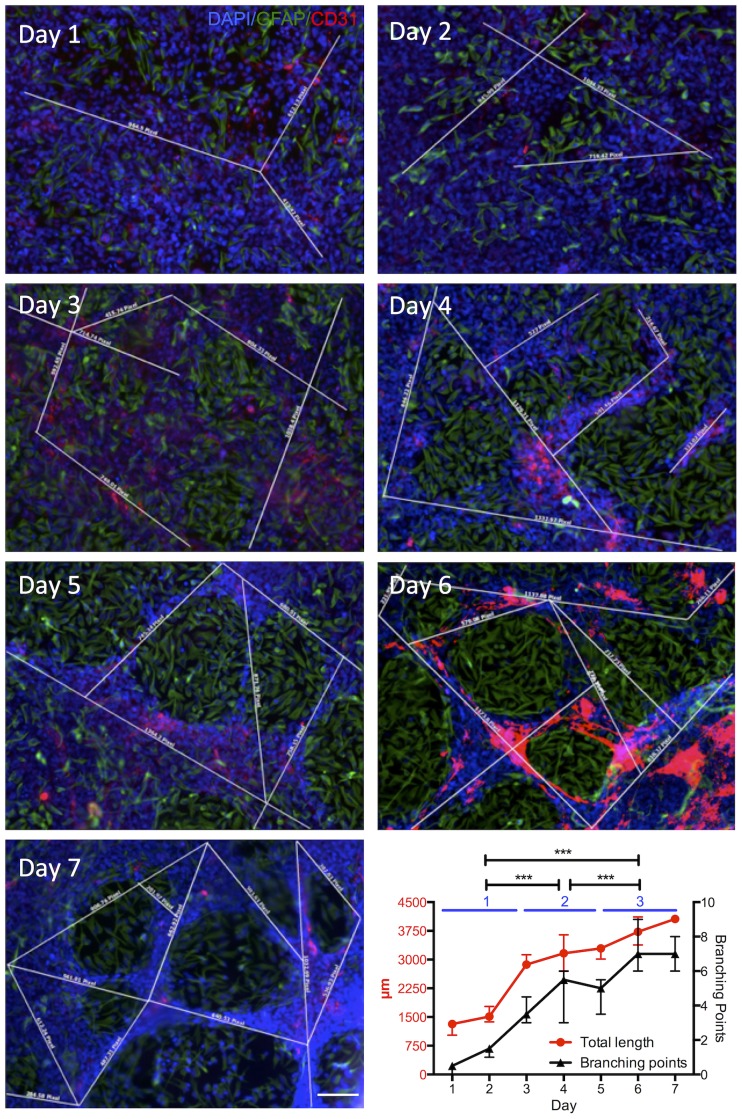
Time course of endothelial morphogenesis. Immunocytochemistry using antibodies against CD31 (red) and polyclonal GFAP (green) revealed vascular morphogenesis in hCMEC/hNSC coculture with different culture durations of 1 to 7 days. The efficiency of endothelial morphogenesis that produced vasculature-like structures, where hCMECs (CD31+) align to form rods, was assessed by measuring the length of these rods (white lines), as well as the number of branching points between these for each image. Based on these quantifications, it was evident that total length of all individual rods and the number of branching points increased over 7 days in a linear fashion. A linear regression allowed the calculation of the slope of this progression and afforded a statistical comparison between both to indicate a significant difference in slope between VLS length and branching points. Diamidino-2-phenylindole (DAPI, blue) serves as a nuclear counterstain. Scale bar, 100 µm. Data points on the graph represent the median with bars reflecting the value range.

A quantification of the VLS by immunohistochemistry over 7 days indicated a gradual process of VLS formation (Figure 10). Initially, no defined VLS were readily identified, although an interaction and movements of hCMECs and hNSCs were evident. Hence the two juxtaposed monolayers gradually interchanged. Clear structural differences between hCMECs and hNSCs emerged that indicated a persisting endothelial morphogenesis in which hNSCs accumulated in patches in between hCMECs and gradually refined the endothelial morphogenesis. Eventually, a well-defined network of VLS was evident with well-defined patches of hNSCs. This gradual organization was also evident in the linear increase in the length of VLS between 1 and 7 days, as well as the number of branches in between these. However, the slope of VLS length and the number of branches was significantly different (F_(1;38)_ = 121.068, p<.001) indicating a stepper increase in the number of branches than the length of segments.

### hCMECs increase neuronal differentiation of hNSCs

**Figure 11:**
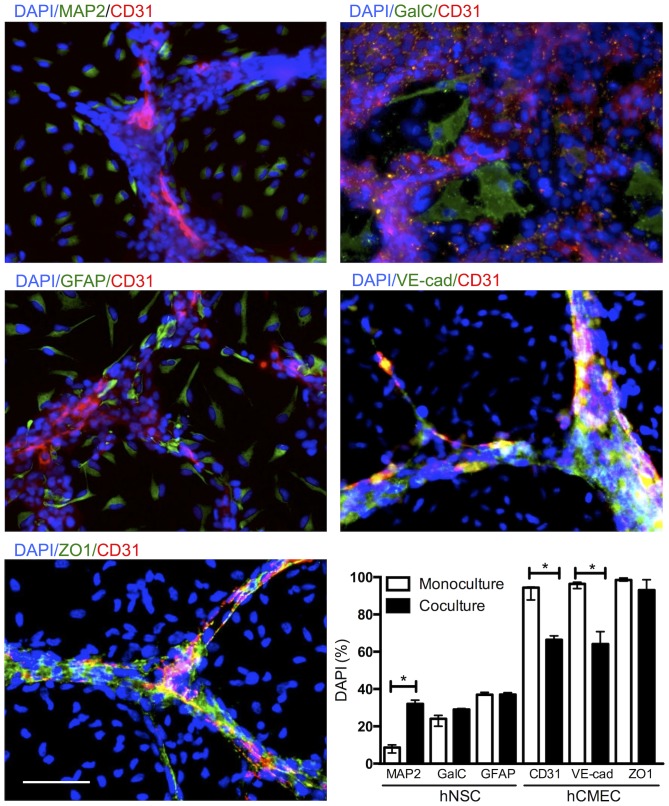
Differentiation status in hCMEC/hNSC coculture. To determine the phenotypic effects of the coculture of hCMECs with hNSCs, specific markers relevant to the differentiation of hNSCs into neurons (MAP2+), astrocytes (monoclonal GFAP+) and oligodendrocytes (GalC+), as well as junction proteins in hCMECs (CD31, VE-cadherin, ZO1) were measured. There was a 3-fold increase in neuronal differentiation in coculture versus monoculture, but no significant effect on astrocytic or oligodendrocyte differentiation. Apart of ZO1, the percentage of hCMECs expressing CD31 and VE-cadherin was significantly reduced in coculture with hNSCs.

Apart of the formation of VLS, coculture of hCMECs with hNSCs also affected their phenotype (Figure 11). Specifically, neuronal differentiation (MAP2+ cells) of hNSCs significantly increased by a factor of 3 in the presence of hCMECs (U = 0, p<0.05), whereas differentiation into oligodendrocytes (GalC+ cells) or astrocytes (GFAP+ cells) was not significantly affected. CD31 (U = 0, p<0.05) and VE-cadherin (U = 0, p<0.05) on hCMECs were downregulated in the presence of hNSCs indicating that the integrity of intercellular junctions was not yet fully restored after 7 days of coculture, although ZO1 was expressed equally in co- and monoculture. The phenotypic consequence of coculturing therefore predominately affected neuronal differentiation for hNSCs, whereas in hCMECs some intercellular junction proteins were reduced reflecting the state of VLS maturation.

## Discussion

The neurovascular unit (NVU) defines the organizational principle of the brain by highlighting the importance of interactions between the vascular and neural compartments [1]. We here present a novel, yet simple, *in vitro* model of human brain neurovascular interactions that affords the investigation of autocrine, paracrine and juxtracrine signaling between neural and endothelial cells during the formation of VLS. Indeed, these cocultures appear to model the neurovascular environment in which VLS form a “vascular network” in between which neural elements form patches of “neural tissue”. This *in vitro* assay will be essential to provide a detailed study of the cellular and molecular interactions between these different cell types to validate or generate novel hypotheses about neovascularization and the NVU in the human brain [34].

### *In vitro* models of the neurovascular environment and the BBB

A key feature of neovascularization is the formation of novel vessel structures from existing blood vessels. In the brain, this process is evident in the case of growing tumors, but also after brain damage, such as stroke, as well as injection of NSCs to promote repair [35]. Although the basic mechanisms of neovascularization in the brain are consistent with vasculogenesis, angiogenesis, arteriogenesis or collateral growth in other organs, important differences exist, specifically in terms of the stabilization of vessels, where astrocytic endfeet directly interact with endothelial cells with tight junctions being created to form the blood-brain barrier [36]. Increasing evidence also suggests that processes involving Wnt signaling and pericytes in the early phases of neurovascular development are crucial for BBB formation [37], [38]. Modeling of the BBB indeed has been the main focus of *in vitro* models to determine its specific characteristics that afford transport of molecules into the brain, a quintessential limitation for delivering therapeutics to the brain by systemic administration [30], [39]–[41].

*In vitro* assays of the BBB, however, are mostly focused on the barrier properties and hence these *in vitro* systems often rely on a single endothelial layer or a dual layer system, where endothelial cells form one layer and astrocytes form a second adjacent layer [42]–[44]. In some cases, a tri-cellular environment is created by adding pericytes to the astrocytes’ cell layer to more closely mimic the cellular environment present at the BBB [22]. The formation of a basement membrane is also considered a crucial characteristic of this environment [44]. These approaches provide useful model systems to study the barrier properties between the endothelial-astrocytes complex, but they do not model the dynamic signaling between vascular and neural elements during vascularization, nor do they model the more complex NVU that also includes neural cells.

The NVU is therefore a more complex structure in which a multitude of cell-cell, cell-matrix and secreted factors from different cell populations interact to form a functional network. Indeed, this complexity is a challenge to examine these interactions *in vivo*. *In vitro* models of cells derived from brain tissue are hence indispensible to thoroughly analyze signaling interactions in the NVU. To this end, it is imperative that not only astrocytes are present in the “neural” compartment, but also neurons and oligodendrocytes that form patches nested in between the vascular tubules forming a network-like structure, as is observed in actual brain tissue [23]. Indeed, after 7 days of coculture, we here observe these features that emerge due to the specific interaction between hCMECs and hNSCs. Astrocytic and oligodendrocyte differentiation was unaffected by endothelial morphogenesis, but neural differentiation increased more than three-fold. Nevertheless, it remains unclear if this is a direct effect of endothelial cell signaling on neural stem cells through the Notch-Delta pathway [45] or if it is indirectly modulated through endothelial cells affecting signaling from astrocytes and oligodendrocytes. Nevertheless, these questions can now be addressed using this *in vitro* assay.

### The formation of vasculature-like structures and vasculogenesis

Understanding the role of vascularization in the adult brain is essential to develop novel therapeutics; be it to block angiogenesis in the case of brain tumors, to enhance neovascularization in the case of stroke, or to promote vascularization of tissue engineered structures [6]. Indeed, vascularization processes, such as vasculogenesis, angiogenesis, arteriogenesis, and collateral growth, share many common molecular signaling pathways, although cellular behavior in these processes is distinct due to their microenvironmental interactions [5], [46]. To this end, it is essential that we develop an understanding of the cellular and molecular processes involved in forming novel vessel structures. The formation of vasculature-like structures (VLS) *in vitro* is a process reflecting vasculogenesis [5].

It is evident here that specific conditions are required *in vitro* for this process to occur. The inability of secreted factors to generate VLS is suggesting that additional components are essential to induce endothelial morphogenesis. Merely adding endothelial and neural cells together, however, to provide juxtacrine, as well as paracrine and autocrine signals, is still insufficient to promote novel vessel structures to form, although endothelial – NSC cocultures have been demonstrated to provide insight into their signaling interactions [19], [45], [47]. For endothelial morphogenesis to occur, ECs and NSCs require specific signaling which is dependent on their differentiation state leading to a cascade of interactions involving autocrine, paracrine and juxtacrine signals.

Specifically, a differentiated endothelium here was a necessary condition for NSCs to induce VLS formation. The requirement of close contact between both types of cells further indicates the involvement of juxtacrine factors as key enablers. Interference with juxtacrine factors, such as the vitronectin receptor α_v_β_3_, prevents angiogenesis [48]. Vitronectin is one of the earliest extracellular matrix proteins deposited in the formation of the basement membrane, providing a motif for NSCs to attach, but also governing interactions between ECs, as they align for endothelial morphogenesis. A variety of additional juxtacrine factors (e.g. laminin) have been identified, but it remains unclear if all these molecules define necessary conditions to induce VLS or if some of these are merely modulating factors that influence the efficiency and urgency of vessel formation [49].

A further complication is the potential synergistic effects of secreted and juxtacrine factors. It remains currently unclear if paracrine, autocrine and juxtacrine factors act as independent “go – no go” signaling points for different processes (e.g. migration, proliferation) or if the combination of these is required to control a specific process. For instance for paracrine factors, it is already known that a combined signaling of transforming growth factor (TGF)-β1 and bFGF is required to stabilize a vessel construct [50]. The specific timing of these factors at a particular site is pivotal to facilitate subsequent steps involved in endothelial morphogenesis [51]. Paracrine signaling might therefore provide the general stimulus for a process (due to its more diffuse nature), but local control over specifically which cells are responsive might be controlled more tightly by juxtacrine factors. An *in vitro* system, as described here, with human cell lines that can be modified to provide “no go” points, as well as administration of blocking agents at different decision points, will be a crucial tool to unravel these processes further.

### Limitations of the assay

It is important to point out that the assay described here is only using human cell lines and hence provides a consistent source of cells to reliably produce VLS embedded within neural compartments. The availability of NSCs from different regions of the human brain [27], [28] further affords modeling of the NVU representing different brain regions. Especially studies investigating hypoxia will benefit from these aspects, as it is known that, for instance, hippocampal cells are more vulnerable than cortical cells [52].

The formation of VLS in hCMEC/hNSC coculture suggests that these two cell types provide the sufficient conditions to produce an endothelial morphogenesis. Nevertheless, it is known that additional cell types, such as pericytes and microglia, populate the NVU in the brain [1]. Specifically, pericytes have been associated with functions of the BBB [53] with measurements of trans-endothelial electrical resistance being required to determine barrier functions [26]. To serve as a “complete” model of the BBB, it would be essential that the formed capillaries can be perfused to determine if indeed a lumen can be formed and if there is transfer of molecules from the vascular to the “neural” compartment [54]. Microglia have also been reported to be involved in the formation of novel blood vessels and are a key modulator of signaling that involves inflammatory cytokines [55]. Therefore to truthfully model the NVU to study, for instance, signaling in hypoxia, it is essential to further expand the assay described here to also include these types of cells.

A general limitation of *in vitro* NVU or vasculogenesis assays are that they are conceived in 2 dimensions for ease of use, as well as analysis. Although the VLS here expanded beyond the monoculture during endothelial morphogenesis, this cannot truly be considered a 3 dimensional environment, but rather a 2D+ culture environment [56]. As the NVU in the brain is a 3D environment, a further advance will be to use, for instance, biomaterials to create an artificial 3D environment *in vitro* [57]. However, care needs to be taken in developing these assays, as biomaterials themselves can provide signaling, as in the case of Matrigel. Eventually these further technical developments will provide more realistic *in vitro* models of vessel formation and the NVU in the human brain.

## Conclusion

*In vitro* models of vessel formation and the NVU using only human cells derived from brain tissues are essential to gain a more thorough understanding of the molecular and cellular processes that govern these in the living human brain. The assay described here relies on human cell lines that afford an abundant availability and high reproducibility. Most importantly, they form vasculature-like structures defined by neural patches containing astrocytes, oligodendrocytes and neurons and provide a model to study neurovascular interactions in vitro. However, there are limitations to this assay, specifically the lack of pericytes and microglia, as well as a 3 dimensional tissue structure, which are key elements of the neurovascular unit. Nevertheless, this assay of a neurovascular environment will provide a useful tool to investigate the dynamic interactions between human endothelial and neural stem cells while forming and maintaining vasculature-like structures.

## Supporting Information

Table S1 **Composition of** **culture media.** Composition of the cell culture media for growing the D3 human cerebral microvascular endothelial cell (hCMEC) line, the STROC05 human neural stem cell (hNSC) line, and the hCMEC/hNSC coculture. Factors with a * were removed to induce cell differentiation.
(PDF)
