# Correction: *In Vitro* Modeling of the Neurovascular Environment by Coculturing Adult Human Brain Endothelial Cells with Human Neural Stem Cells

**DOI:** 10.1371/journal.pone.0117650

**Published:** 2015-01-30

**Authors:** The PLOS ONE Staff

The images for Figs. 3 and 4 are incorrectly switched. The image that appears as Fig. 3 should be Fig. 4, and the image that appears as Fig. 4 should be Fig. 3. The figure legends appear in the correct order.

**Figure 3:**
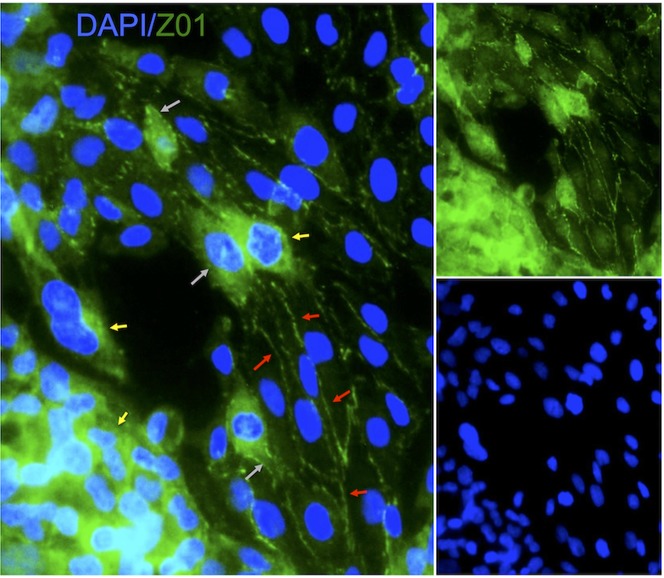
Immunohistochemistry of junctional markers on endothelial cells. Localization of ZO1 expression/detection was heterogeneous, with it being only visible as part of the membrane (i.e. cellular junctions) on elongated and arranged endothelial cells (red arrows), but being mostly cytoplasmic in clumped and non-elongated cells (yellow arrows). As the cytoplasmic expression is decreased (grey arrow), it gradually shifts towards being exclusively present in the membrane.

**Figure 4:**
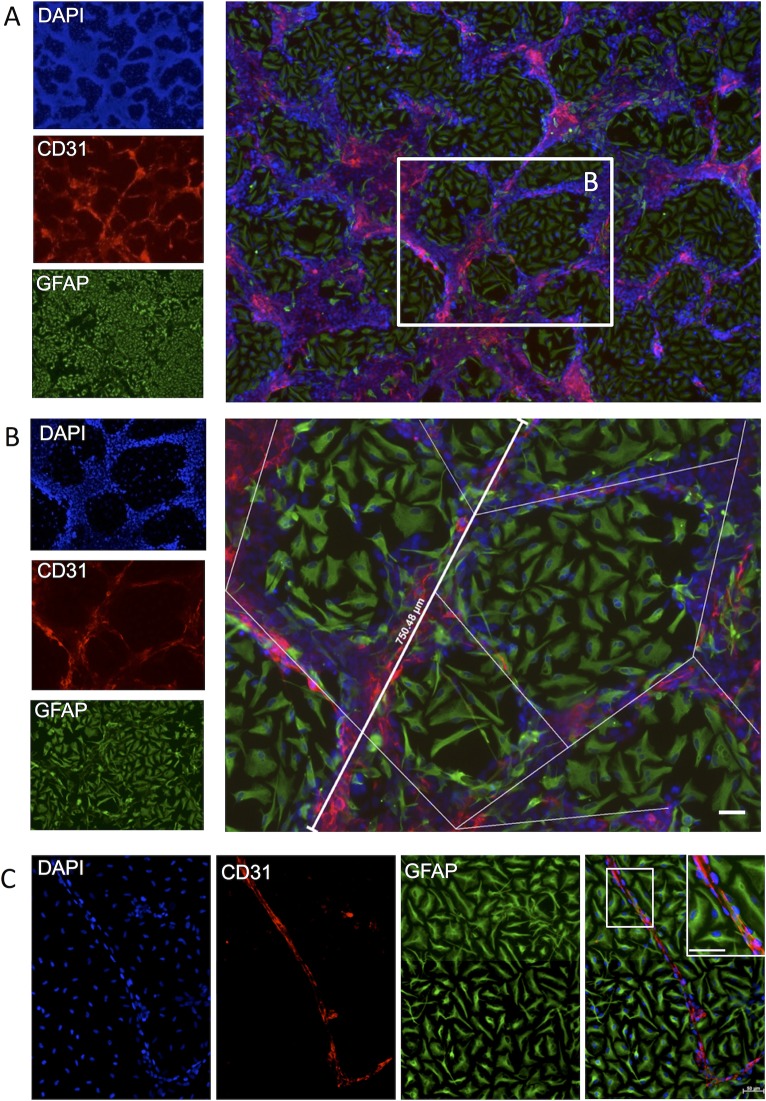
Quantification of endothelial morphogenesis. (A) A distinctive neurovascular cytoarchitecture emerged in which hCMECs (CD31+) formed vasculature-like structures (VLS) resembling a vascular network in between patches of hNSCs (polyclonal GFAP+). (B) The efficiency of VLS formation was quantified by measuring the length of segments between VLS branching points. (C) In a few samples, singular capillary-like structures comprising single layers of CD31+ cells between which a lumen-like space formation was observed. Diamidino-2-phenylindole (DAPI, blue) serves as a nuclear counterstain. Scale bars represent 50 μm.

There is an error in Fig. 10. The authors have provided the corrected figure below.

**Figure 10:**
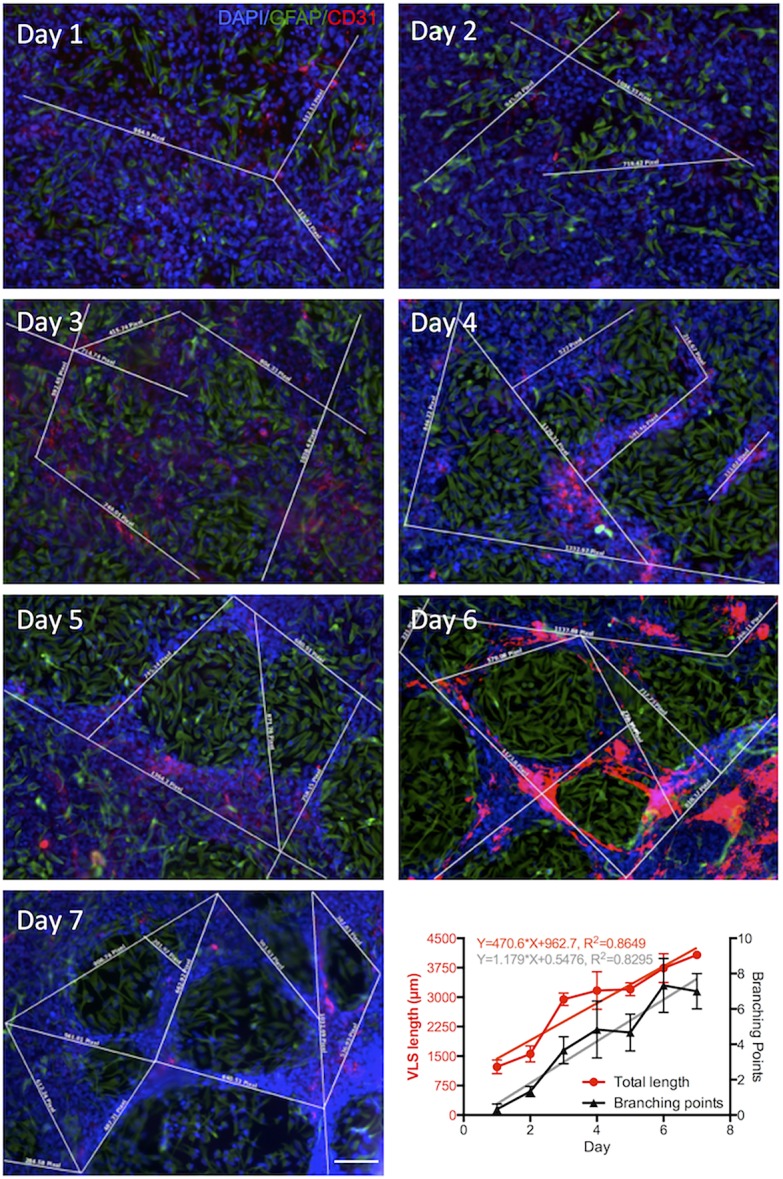
Time course of endothelial morphogenesis. Immunocytochemistry using antibodies against CD31 (red) and polyclonal GFAP (green) revealed vascular morphogenesis in hCMEC/hNSC coculture with different culture durations of 1 to 7 days. The efficiency of endothelial morphogenesis that produced vasculature-like structures, where hCMECs (CD31+) align to form rods, was assessed by measuring the length of these rods (white lines), as well as the number of branching points between these for each image. Based on these quantifications, it was evident that total length of all individual rods and the number of branching points increased over 7 days in a linear fashion. A linear regression allowed the calculation of the slope of this progression and afforded a statistical comparison between both to indicate a significant difference in slope between VLS length and branching points. Diamidino-2-phenylindole (DAPI, blue) serves as a nuclear counterstain. Scale bar, 100 μm. Data points on the graph represent the median with bars reflecting the value range.
