# Supplementary material for: In Vitro Modeling of the Neurovascular Environment by Coculturing Adult Human Brain Endothelial Cells with Human Neural Stem Cells
Source: PLoS One. 2014 Sep 4;9(9):e106346. doi: 10.1371/journal.pone.0106346 (PMC4154686; doi:10.1371/journal.pone.0106346)
Supplement: Table S1 — Composition of culture media. Composition of the cell culture media for growing the D3 human cerebral microvascular endothelial cell (hCMEC) line, the STROC05 human neural stem cell (hNSC) line, and the hCMEC/hNSC coculture. Factors with a * were removed to induce cell differentiation. (PDF) [file pone.0106346.s001.pdf]

Component	EC	NSC	EC/NSC	Supplier	Cat. Ref.
DMEM:F12		Basal medium	Basal medium (half volume)	Sigma-Aldrich	D6421
EBM-2	Basal medium		Basal medium (half volume)	Lonza	CC-3156
Fetal bovine serum	5%			PAA	A15-151
Hydrocortisone	1.4 µM		0.7 µM	Sigma-Aldrich	H-0135
Acid ascorbic	5 µg/ml		2.5 µg/ml	Sigma-Aldrich	A4544
Chemically defined lipid concentrate	1%		0.5%	Invitrogen	11905-031
HEPES	10 mM		5 mM	Sigma-Aldrich	83264
Penicillin/ Streptomycin	1%		0.5%	Invitrogen	15140-122
Human albumin solution		0.03%	0.015%	GemBio	800-121
Transferrin, human		100 µg/ml	50 µg/ml	Sigma-Aldrich	T1147
Putrescine DiHCl		16.2 µg/ml	8.1 µg/ml	Sigma-Aldrich	P5780
Insulin, human		5 µg/ml	2.5 µg/ml	Sigma-Aldrich	I9278
Progesterone		60 ng/ml	30 ng/ml	Sigma-Aldrich	P8783
L-glutamine		2 mM	1 mM	Sigma-Aldrich	G7513
Sodium selenite		40 ng/ml	20 ng/ml	Sigma-Aldrich	S9133
L-thyroxine (T4)		400 ng/ml	200 ng/ml	Sigma-Aldrich	T0397
Tri-iodo-thyronine (T3)		337 ng/ml	118.5 ng/ml	Sigma-Aldrich	T6397
Heparin sodium		10 Units/ml	5 Units/ml	Sigma-Aldrich	H3149
Corticosterone		40 ng/ml	20 ng/ml	Sigma-Aldrich	C2505
*bFGF		10 ng/ml		PeproTech	AF-100-18B
*EGF		20 ng/ml		PeproTech	AF-100-15
*4-hydroxytamoxifen		100 nM		Sigma-Aldrich	H7904

Table S1. Composition of culture media. Composition of the cell culture media for growing the D3 human cerebral microvascular endothelial cell (hCMEC) line, the STROC05 human neural stem cell (hNSC) line, and the hCMEC/hNSC coculture. Factors with a * were removed to induce cell differentiation.
